# XELOX (capecitabine plus oxaliplatin) plus bevacizumab (anti-VEGF-A antibody) with or without adoptive cell immunotherapy in the treatment of patients with previously untreated metastatic colorectal cancer: a multicenter, open-label, randomized, controlled, phase 3 trial

**DOI:** 10.1038/s41392-024-01788-2

**Published:** 2024-04-03

**Authors:** Qiu-Zhong Pan, Jing-Jing Zhao, Liang Liu, Dong-Sheng Zhang, Li-Ping Wang, Wen-Wei Hu, De-Sheng Weng, Xiang Xu, Yi-Zhuo Li, Yan Tang, Wei-Hong Zhang, Jie-Yao Li, Xiao Zheng, Qi-Jing Wang, Yong-Qiang Li, Tong Xiang, Li Zhou, Shuang-Ning Yang, Chen Wu, Rong-Xing Huang, Jia He, Wei-Jiao Du, Lu-Jun Chen, Yue-Na Wu, Bin Xu, Qiong Shen, Yi Zhang, Jing-Ting Jiang, Xiu-Bao Ren, Jian-Chuan Xia

**Affiliations:** 1 State Key Laboratory of Oncology in South China, Guangdong Provincial Clinical Research Center for Cancer, Collaborative Innovation Center for Cancer Medicine, Sun Yat-Sen University Cancer Center, Guangzhou, Guangdong 510060 PR China; 2 Department of Biotherapy, Sun Yat-Sen University Cancer Center, Guangzhou, Guangdong 510060 PR China; 3 Tianjin Medical University Cancer Institute and Hospital, National Clinical Research Center for Cancer, Tianjin, 300060 PR China; 4 Key Laboratory of Cancer Immunology and Biotherapy, Tianjin, 300060 PR China; 5 Department of Biotherapy/Immunology, Tianjin Medical University Cancer Institute and Hospital, Tianjin, 300060 PR China; 6 Department of Medical Oncology, Sun Yat-sen University Cancer Center, Guangzhou, 510060 PR China; 7 Department of Oncology, the First Affiliated Hospital of Zhengzhou University, Zhengzhou, 450052 PR China; 8 Department of Tumor Biological Treatment, the Third Affiliated Hospital of Soochow University, Changzhou, Jiangsu 213003 PR China; 9 Jiangsu Engineering Research Center for Tumor Immunotherapy, Changzhou, Jiangsu 213003 PR China; 10 Institute for Cell Therapy of Soochow University, Changzhou, Jiangsu 213003 PR China; 11 Department of Stem Cell & Regenerative Medicine, State Key Laboratory of Trauma, Burn and Combined Injury, Daping Hospital, Army Medical University, Chongqing, 400042 PR China; 12 Imaging Diagnosis and Interventional Center, Sun Yat-sen University Cancer Center, Guangzhou, Guangdong 510060 PR China

**Keywords:** Gastrointestinal cancer, Cancer therapy

## Abstract

Fluoropyrimidine-based combination chemotherapy plus targeted therapy is the standard initial treatment for unresectable metastatic colorectal cancer (mCRC), but the prognosis remains poor. This phase 3 trial (ClinicalTrials.gov: NCT03950154) assessed the efficacy and adverse events (AEs) of the combination of PD-1 blockade-activated DC-CIK (PD1-T) cells with XELOX plus bevacizumab as a first-line therapy in patients with mCRC. A total of 202 participants were enrolled and randomly assigned in a 1:1 ratio to receive either first-line XELOX plus bevacizumab (the control group, *n* = 102) or the same regimen plus autologous PD1-T cell immunotherapy (the immunotherapy group, *n* = 100) every 21 days for up to 6 cycles, followed by maintenance treatment with capecitabine and bevacizumab. The main endpoint of the trial was progression-free survival (PFS). The median follow-up was 19.5 months. Median PFS was 14.8 months (95% CI, 11.6–18.0) for the immunotherapy group compared with 9.9 months (8.0–11.8) for the control group (hazard ratio [HR], 0.60 [95% CI, 0.40–0.88]; *p* = 0.009). Median overall survival (OS) was not reached for the immunotherapy group and 25.6 months (95% CI, 18.3–32.8) for the control group (HR, 0.57 [95% CI, 0.33–0.98]; *p* = 0.043). Grade 3 or higher AEs occurred in 20.0% of patients in the immunotherapy group and 23.5% in the control groups, with no toxicity-associated deaths reported. The addition of PD1-T cells to first-line XELOX plus bevacizumab demonstrates significant clinical improvement of PFS and OS with well tolerability in patients with previously untreated mCRC.

## Introduction

Colorectal cancer (CRC) is a common and aggressive cancer globally, ranking as the third most frequently new diagnosed cancer.^1^ In 2020, there were over 1.9 million new cases of CRC reported worldwide, with more than 935,000 deaths.^1^ Although radical surgery remains the optimal treatment for non-metastatic CRC, about one-third of patients experience tumor relapse with eventual distant metastases over the course of the disease.^2^ Due to the lack of clear clinical symptoms and signs in early stages, around 20% of patients who are newly diagnosed with CRC have distant metastases.^3^ Fluoropyrimidine-based combination chemotherapy plus targeted therapy is currently recommended initial treatment for metastatic CRC (mCRC). Among various chemotherapy regimens, XELOX (capecitabine plus oxaliplatin) with or without bevacizumab regimens is one of the optional first‐line treatments of mCRC. However, the clinical benefits of XELOX plus bevacizumab regimens remain limited, resulting in a median progression-free survival (PFS) of about 10 months and a median overall survival (OS) ranging from 21 to 26 months.^4–7^ The addition of other targeted drug (such as cetuximab) to XELOX and bevacizumab cannot further improve the survival,^8^ suggesting the conventional treatment of mCRC has reached a plateau and there is urgent clinical need for developing novel therapies for patients with mCRC.

In recent years, immune checkpoint blockade (ICB) therapy has revolutionized the landscape for the management of many solid tumors due to their superior efficacy. ICB therapy is highly recommended for mCRC patients characterized by the DNA mismatch repair–deficient (dMMR)/microsatellite instability-high (MSI-H).^3,9^ In contrast, either single-agent ICB therapy or ICB in combination with first-line chemotherapy plus bevacizumab did not show a significant advantage in PFS for patients with proficient MMR (pMMR) mCRC,^10,11^ which accounts for 95% of patients with mCRC.^9^ Adoptive cell immunotherapy presents a potential alternative immunotherapeutic approach for mCRC patients by administering immune active cells.^12–14^ Currently, several kinds of immune active cells, such as cytokine‐induced killer cells (CIK)/dendritic cells–co‐cultured CIK cells (DC‐CIK), tumor infiltrating lymphocytes (TILs), chimeric antigen receptor T cells (CAR-T), T cell receptor T cells (TCR-T), and natural killer cells (NK), have been extensively investigated in the context of CRC.^14^ Among them, CIK/DC-CIK cells have shown promising outcomes in initial clinical trials and demonstrated synergistic anti-tumor effects when coadministered with conventional cancer therapy.^13,15^

Our previous studies have demonstrated that adoptive CIK cell immunotherapy combining with first‐line chemotherapy significantly enhances the survival outcomes in patients with mCRC.^16,17^ A recent meta-analysis involving 6743 patients from 70 studies also supports this finding, showing that adoptive CIK/DC-CIK cell immunotherapy in combination with standard treatment protocols, particularly chemotherapy, offers remarkable clinical benefits for patients with CRC.^13^ However, there remains a lack of randomized, controlled, multicenter clinical studies to further confirm the therapeutic efficacy of adoptive immune cell therapy in CRC.

To overcome the immune barrier of the tumor microenvironment, we developed a novel PD‐1 blockade‐activated DC-CIK cells (hereafter referred to as PD1-T cells), which were manufactured by blocking the PD‐1 epitope in DC-CIK cells with a low-dose of anti-PD-1 antibody (pembrolizumab).^18,19^ Our previous clinical trials indicated a good safety and efficacy for PD1-T cells in solid tumor.^18,19^ Based on these findings, we conducted a randomized, multicenter, phase 3 trial to evaluate whether the addition of PD1-T cells to XELOX plus bevacizumab improves efficacy compared to XELOX plus bevacizumab alone in patients with previously untreated mCRC.

## Results

### Patient and treatment characteristics

**Fig. 1:**
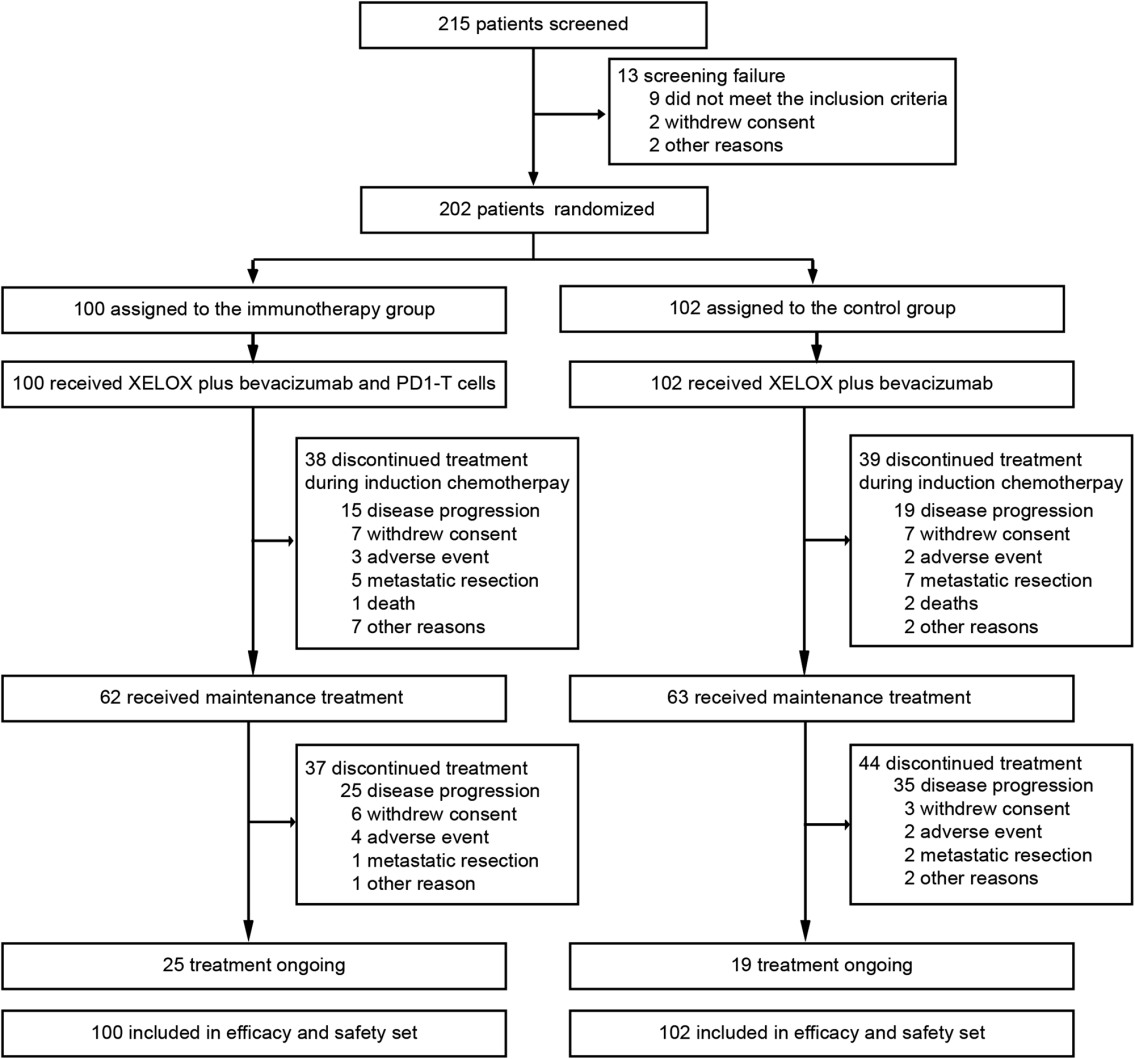
Trial profile. XELOX, capecitabine and oxaliplatin; PD1-T cells, PD‐1 blockade‐activated DC-CIK cells

Between March 20, 2019 and April 7, 2022, 215 patients with mCRC were assessed for eligibility. Among them, 202 were randomly assigned in a 1:1 ratio, including 100 and 102 in the immunotherapy and the control groups, respectively (Fig. 1). The baseline demographic and clinical characteristics showed no significant differences between the 2 treatment arms (Table 1). The cutoff date for the analyses was September 9, 2022. The median follow-up time was 19.5 months in the whole cohort. All participants who underwent randomization (*N* = 202) received at least one dose of the designated study treatment and were included in the both intention-to-treat analysis set and safety population (Fig. 1).

**Table 1:** Demographic and patient characteristics at baseline

Characteristics, *n* (%)	All patients (*n* = 202)	Immunotherapy group (*n* = 100)	Control group (*n* = 102)	*P*
Sex				0.068
Male	104 (51.5%)	45 (45.0%)	59 (57.8%)	
Female	98 (48.5%)	55 (55.0%)	43 (42.2%)	
Age				0.252
< 60	111 (55.0%)	59 (59.0%)	52 (51.0%)	
≥60	91 (45.0%)	41 (41.0%)	50 (49.0%)	
ECOG performance status				0.574
0	6 (3.0%)	3 (3.0%)	3 (2.9%)	
1	192 (95.0%)	94 (94.0%)	98 (96.1%)	
2	4 (2.0%)	3 (3.0%)	1 (1.0%)	
Primary tumor site				0.454
Left-sided	122 (60.4%)	63 (63.0%)	59 (57.8%)	
Right-sided	80 (39.6%)	37 (37.0%)	43 (42.2%)	
Metastatic sites				0.691
Liver	136 (67.3%)	66 (66.0%)	70 (68.6%)	
Liver not affected	66 (32.7%)	34 (34.0%)	32 (31.4%)	
Number of metastatic sites				0.489
1	90 (44.6%)	47 (47.0%)	43 (42.2%)	
≥2	112 (55.4%)	53 (53.0%)	59 (57.8%)	
Previous adjuvant chemotherapy				0.488
No	177 (87.6%)	86 (86.0%)	91 (89.2%)	
Yes	25 (12.4%)	14 (14.0%)	11 (10.8%)	
RAS status				0.734
Wild-type	98 (48.5%)	51 (51.0%)	47 (46.1%)	
Mutant	76 (37.6%)	35 (35.0%)	41 (40.2%)	
Missing data	28 (13.9%)	14 (14.0%)	14 (13.7%)	
BRAF status				0.859
Wild-type	157 (77.7%)	79 (79.0%)	78 (76.5%)	
Mutant	14 (6.9%)	6 (6.0%)	8 (7.8%)	
Missing data	31 (15.3%)	15 (15.0%)	16 (15.7%)	
Mismatch repair status				0.945
Proficient	173 (85.6%)	85 (85.0%)	88 (76.3%)	
Deficient	5 (2.5%)	3 (3.0%)	2 (2.0%)	
Missing data	24 (11.9%)	12 (12.0%)	12 (11.8%)	

Control group, XELOX plus bevacizumab; Immunotherapy group, XELOX plus bevacizumab and PD-1 blocked-activated DC-CIK cells

*ECOG* Eastern Cooperative Oncology Group

During the induction therapy phase, both groups were administered a median six cycles of XELOX plus bevacizumab (range, 1–6 cycles). The median cumulative doses during the induction phase were similar between the two treatment groups (supplementary Table 1). A total of 62 (62.0%) patients in the immunotherapy group and 63 (61.8%) in the control group continued to receive maintenance therapy. By the cutoff date, the immunotherapy group had 25 (25.0%) patients and the control group had 19 (18.6%) patients who were still on the study treatment (Fig. 1). Among the reasons leading to treatment discontinuation, disease progression was identified as the primary factor: 40 (40.0%) patients discontinued in the immunotherapy group compared to 54 (52.9%) in the control group (Fig. 1). A significant proportion of patients who discontinued the study treatment opted for further anti-cancer therapy: specifically, 74.7% (56 out of 75) in the immunotherapy group and 79.5% (66 out of 83) in the control group (supplementary Table 2).

Autologous PD1-T cells were successfully cultured in all 100 patients of the immunotherapy group. After 2 weeks of culture, the median count of PD1-T cells was 1.2 × 10^10^ (range, 0.6 × 10^10^ to 1.9 × 10^10^) for all cycles. The phenotype of final PD1-T cell products was shown in the supplementary Table 3. Except for one patient who died of obstructive pneumonia due to lung metastases after the first cycle of chemotherapy and did not receive any PD1-T cell infusion, all other patients received at least one cycle of PD1-T cell infusion. Of these, 83% received four or more cycles of PD1-T cell agent and 70% completed six cycles of PD1-T cell agent (supplementary Table 3). The mean number of transferred PD1-T cells was 1.2 × 10^10^ (SD, 0.2) per cycle and 6.1 × 10^10^ (SD, 2.2) per patient (supplementary Table 3).

### Efficacy

**Fig. 2:**
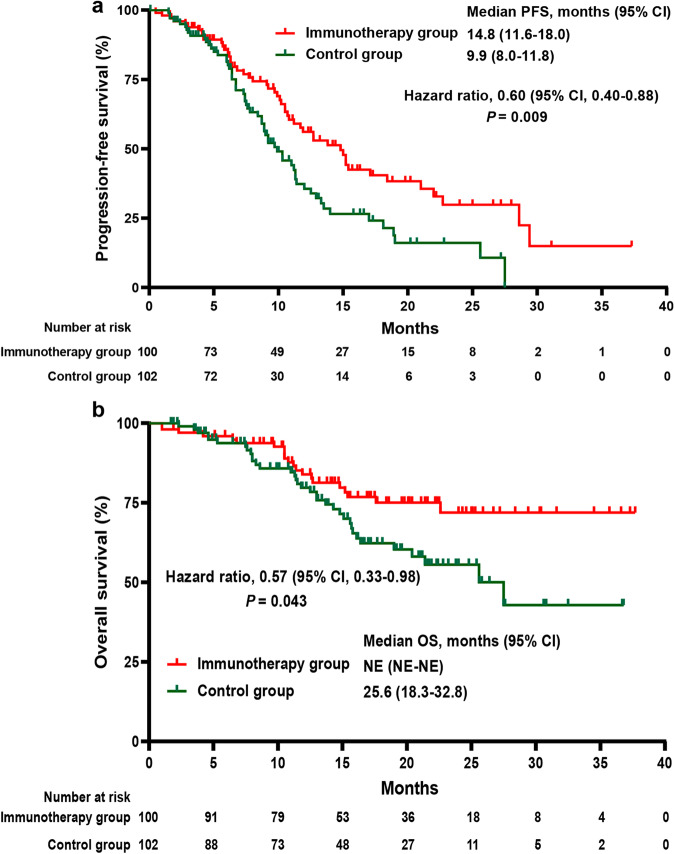
Progression-free survival (PFS) and overall survival (OS) of patients in the two treatment groups. **a** Kaplan-Meier estimates of PFS in the intention-to-treat population. **b** Kaplan-Meier estimates of OS in the intention-to-treat population. Crosses denote censored patients. Control group, XELOX plus bevacizumab; Immunotherapy group, XELOX plus bevacizumab and PD-1 blocked-activated DC-CIK cells

**Fig. 3:**
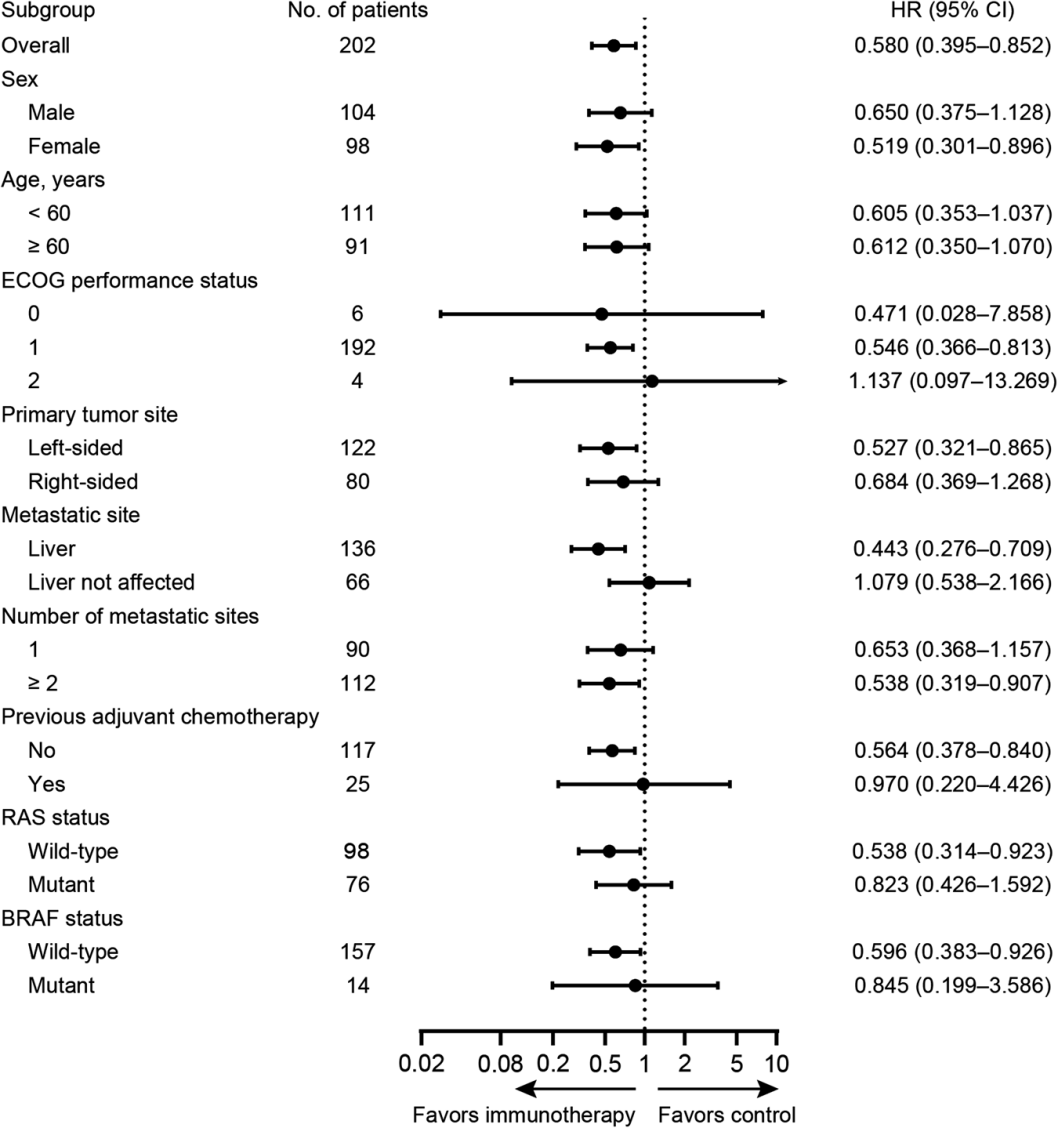
Subgroup analyses of progression-free survival according to clinical and molecular characteristics. All hazard ratios (HR) were computed using the Cox proportional hazards model

At data cutoff, 50 (50.0%) patients in the immunotherapy group experienced disease progression or death, as had 60 (58.8%) patients in the control group. This study successfully achieved its primary endpoint by showing a median PFS of 14.8 months (95% confidence interval [CI], 11.6–18.0) in the immunotherapy group, as compared with 9.9 months (95% CI, 8.0–11.8) in the control group (stratified Hazard Ratio [HR], 0.60; 95% CI, 0.40–0.88; stratified log-rank *p* = 0.009; Fig. 2a). Post hoc sensitivity analyses suggested that the treatment effect exhibited in the study was robust, and treatment discontinuations did not unduly influence the conclusion of the primary PFS analysis (Supplementary Table 4). Further sensitivity analyses revealed that the effect of study treatment on PFS remained consistent regardless of the RAS or BRAF mutation status, as well as MMR status of the tumor. Interaction tests for treatment and both RAS/BRAF mutation status and MMR status yielded negative results in terms of PFS (Supplementary Table 5). Exploratory post­hoc subgroup analyses for PFS suggested a benefit from the PD1-T cell immunotherapy combined with XELOX plus bevacizumab in all subgroups apart from patients with ECOG performance status (PS) of 2 and those without liver metastases, although many of the results didn’t reach the statistically significant (Fig. 3). For patients with pMMR tumors, the immunotherapy group exhibited a significantly reduced risk of disease progression or death compared to the control group, with a hazard ratio (HR) of 0.62 (95% CI, 0.41–0.94) (supplementary Fig. 1).

At data cutoff, 56 (27.7%) patients had died: 21 (21.0%) patients in the immunotherapy group and 35 (34.3%) patients in the control group. Median overall survival was not reached for patients receiving PD1-T cell immunotherapy combined with XELOX plus bevacizumab, while it was 25.6 months (95% CI, 18.3–32.8) for patients receiving XELOX plus bevacizumab alone (stratified HR, 0.57, 95% CI, 0.33–0.98; stratified log-rank *p* = 0.043; Fig. 2b).

According to the RECIST (version 1.1) criteria, as evaluated by independent radiologists, confirmed complete response was achieved in 3 (3.0%) patients from the immunotherapy group and 2 (2.0%) from the control group (Table 2). Additionally, confirmed partial response was observed in 51 (51.0%) patients from the immunotherapy group and 46 (45.1%) from the control group (Table 2). 7 out of 202 patients (3.5%) did not undergo radiographic evaluation. The immunotherapy and the control groups did not show significant differences in overall response rate (ORR) (54.0% versus 47.1%, *p* = 0.324) or disease control rate (DCR) (93.0% versus 96.1%, *p* = 0.335). The immunotherapy group had a median duration of response (DOR) of 13.4 months (95% CI, 9.8–17.0), while the control group had a DOR of 7.0 months (95% CI, 5.9–8.1). The hazard ratio for DOR between the two groups was 0.50 (95% CI, 0.29–0.86; *p* = 0.011; Table 2).

**Table 2:** Tumor response in patients of the two treatment groups

Variable	Immunotherapy group (*n* = 100)	Control group (*n* = 102)	*P*
Efficacy			
ORR	54	48	0.324
% (95% CI)	54.0 (43.7–64.0)	47.1 (37.1–57.2)	
DCR	93	98	0.335
% (95% CI)	93.0 (86.1–97.1)	96.1 (90.3–98.9)	
Best response, *n* (%)			
CR	3 (3.0%)	2 (2.0%)	
PR	51 (51.0%)	46 (45.1%)	
SD	39 (39.0%)	50 (49.0%)	
PD	2 (2.0%)	2 (2.0%)	
NE	5 (5.0%)	2 (2.0%)	
Median DOR, months (95% CI)	13.4 (9.8–17.0)	7.0 (5.9–8.1)	0.011

Control group, XELOX plus bevacizumab; Immunotherapy group, XELOX plus bevacizumab and PD-1 blocked-activated DC-CIK cells

*ORR* objective response rate, *DCR* disease control rate, *CR* complete response, *PR* partial response, *SD* stable disease, *PD* progressive disease, *NE* not evaluated, *DOR* duration of response

### Safety

The safety analysis cohort comprised all 202 patients who underwent study treatment. 94.0% of patients in the immunotherapy group and 96.1% of patients in the control group experienced treatment-related adverse events (TRAEs) (Table 3). The incidence of grade 3 or more severe adverse events was comparable in both groups, with a frequency of 20 of 100 (20.0%) patients in the immunotherapy group and 24 of 102 (23.5%) patients in the control group (Table 3). The most common grade 3 or more severe events in the immunotherapy group compared to the control group were leukopenia (6.0% versus 4.9%), hand-foot syndrome (4.0% versus 3.9%), anemia (3.0% versus 2.0%), allergy (3.0% versus 2.9%), diarrhea (2.0% versus 4.9%), thrombocytopenia (2.0% versus 3.9%), gastrointestinal obstruction (2.0% versus 2.0%) and oral mucositis (1.0% versus 2.0%). Serious adverse events were observed in 13 (13.0%) patients receiving immunotherapy and in 15 (14.7%) patients from the control group (supplementary Table 6). No deaths related to toxicity were observed in this study. Treatment discontinuation due to TRAEs was observed in 7 patients (7.0%) in the immunotherapy group and 4 (4.0%) in the control group.

**Table 3:** Treatment-related adverse events

Adverse event	Immunotherapy group (*n* = 100)		Control group (*n* = 102)	
	Any grade	≥ Grade 3	Any grade	≥ Grade 3
Any event	94 (94.0%)	20 (20.0%)	98 (96.1%)	24 (23.5%)
Anemia	57 (57.0%)	3 (3.0%)	55 (53.9%)	2 (2.0%)
Leukopenia	54 (54.0%)	6 (6.0%)	54 (52.9%)	5 (4.9%)
Nausea	50 (50.0%)	1 (1.0%)	48 (47.1%)	0
Fatigue	42 (42.0%)	0	34 (33.3%)	0
AST increased	39 (39.0%)	0	32 (31.4%)	1 (1.0%)
ALT increased	36 (36.0%)	0	28 (27.5%)	1 (1.0%)
Vomiting	35 (35.0%)	1 (1.0%)	39 (38.2%)	0
Thrombocytopenia	28 (28.0%)	2 (2.0%)	35 (34.3%)	4 (3.9%)
Hypoproteinemia	28 (28.0%)	0	25 (24.5%)	0
Peripheral neurotoxicity	26 (26.0%)	0	30 (29.4%)	0
Hand-foot syndrome	21 (21.0%)	4 (4.0%)	19 (18.6%)	4 (3.9%)
Diarrhea	20 (20.0%)	2 (2.0%)	25 (24.5%)	5 (4.9%)
Proteinuria	20 (20.0%)	0	26 (24.5%)	0
Oral mucositis	18 (18.0%)	1 (1.0%)	13 (12.7%)	2 (2.0%)
Constipation	16 (16.0%)	0	15 (14.7%)	0
Hyperbilirubinemia	15 (15.0%)	1 (1.0%)	16 (15.7%)	0
Abdominal pain	13 (13.0%)	0	8 (7.9%)	0
TSH increased	11 (11.0%)	0	13 (12.7%)	0
Bleeding	11 (11.0%)	0	11 (10.8%)	0
Allergy	10 (10.0%)	3 (3.0%)	6 (5.9%)	3 (2.9%)
Hypertension	7 (7.0%)	0	7 (6.9%)	1 (1.0%)
Pyrexia	7 (7.0%)	0	6 (5.9%)	0
Hypokalemia	6 (6.0%)	0	8 (7.9%)	0
Decreased appetite	5 (5.0%)	0	9 (8.8%)	0
Gastrointestinal obstruction	5 (5.0%)	2 (2.0%)	3 (2.9%)	2 (2.0%)
Hypocortisolism	3 (3.0%)	0	2 (2.0%)	0
Hyperthyroidism	3 (3.0%)	0	0	0
Thrombosis	2 (2.0%)	0	0	0
Hyponatremia	2 (2.0%)	0	7 (6.9%)	0
Hypothyroidism	1 (1.0%)	0	0	0
Pneumonia	1 (1.0%)	1 (1.0%)	0	0

Control group, XELOX plus bevacizumab; Immunotherapy group, XELOX plus bevacizumab and PD-1 blocked-activated DC-CIK cells

*AST* aspartate aminotransferase, *ALT* alanine aminotransferase, *THS* thyroid stimulating hormone

The adverse events associated with PD1-T cell agent were typically of grade 1–2 severity, which included fatigue (6, 6.0%), pyrexia (2, 2.0%), hypocortisolism (1, 1.0%), hypothyroidism (1, 1.0%), and hyperthyroidism (3, 3.0%). One patient in the immunotherapy experienced grade 3 pneumonia event, which was considered to have a remote, possible, or probable relationship with PD1-T cell therapy. Five patients discontinued the PD1-T cell treatment for the following reasons (one patient for each): pneumonia, disease progression, withdrew consent, hyperbilirubinemia, and gastrointestinal obstruction.

## Discussion

In this phase 3 trial conducted across multiple centers, we have demonstrated that the combination of PD1-T cells with XELOX plus bevacizumab leads to a significant improvement in both progression-free survival and overall survival when compared to the use of XELOX plus bevacizumab alone in patients with metastatic colorectal cancer. There were no notable variances observed in terms of ORR and DCR, and the addition of PD1-T cells did not raise any new safety concerns.

At the time of planning the trial, immune checkpoint inhibitors had not yet been approved as the first-line treatment for the dMMR mCRC, so patients were not mandatory to detect the MMR status during screening, and patients with dMMR mCRC were also included in the trial. Based on the available MMR status of the enrolled patients, the distribution of patients with dMMR was balance between the two arms. Besides, no significant statistical interaction was observed between the treatment effect and MMR status for PFS. Thus, a potential confounding effect on treatment outcome associated with the difference in MMR status can be excluded.

In this study, the combination of XELOX and bevacizumab was selected as the initial therapy for both dMMR and pMMR mCRC according to the recommendations from global and regional guidelines.^3^ After induction and maintenance treatment, few patients went to receive surgery. This finding suggests that patients were correctly selected to participate in the trial, as eligible patients should have been considered to be inappropriate for surgery according to the study design.

In recent years, adoptive cell immunotherapy has mainly focused on gene engineered T cell therapy, such as CAR-T cells, and TCR-T cells. However, the application of gene engineered T cell therapy in mCRC is limited in regards to efficacy and toxicity.^20,21^ In contrast, previous studies have attempted to investigate the impact of combining chemotherapy with CIK/DC-CIK cell immunotherapy in patients with mCRC, and the findings indicated that the addition of CIK/DC-CIK cells to chemotherapy significantly improved clinical outcomes compared with chemotherapy alone.^13,22^ Our previous phase II study also found that CIK cell therapy combined with 5-fluorouridine, leucovorin and oxaliplatin (FOLFOX4) chemotherapy significantly prolonged OS when compared to the use of chemotherapy alone as an initial treatment for patients with mCRC; while the PFS was not significantly improved.^17^ The current study demonstrated that PD1-T cells combined with XELOX plus bevacizumab exhibited superior efficacy in terms of PFS and OS compared to XELOX plus bevacizumab alone. The difference in the impact of adoptive cell immunotherapy on PFS of patients with mCRC observed in our current and previous studies might be attributed to variations in trial design, chemotherapeutic regimes and immune cell type. In our previous study, the primary endpoint focused on 3-year OS rate, and patients received CIK cell treatment combined with FOLFOX4 regimen without bevacizumab.^17^ However, in the current study, the primary endpoint was PFS, and patients received PD-1 blockade-activated DC-CIK cell treatment combined with XELOX plus bevacizumab. Therefore, the previous study may be not sufficiently powered to detect differences in PFS as the sample size was computed with respect to the primary endpoint of 3-year OS rate. Besides, chemotherapy without bevacizumab and the use of immune cells without anti-PD-1 antibody activation in the previous study may also affect the potential differences in PFS between the treatment groups. Nonetheless, these findings together provide compelling evidence in favor of the effectiveness of combining adoptive cell therapy with first‐line chemotherapy for improved outcomes of patients with mCRC.

One of the obstacles of immune cell therapy is the presence of an immunosuppressive tumor microenvironment.^23^ PD-1/PD-L1 inhibitory signal is the thoroughly studied immunosuppressive pathway, and antibodies targeting PD-1/PD-L1 have gained approval for treating various types of tumors. However, systemic therapy with anti–PD‐1 antibody has been correlated with immune‐related side effects and may trigger an immunosuppressive response through interacting with different types of immunosuppressive cells present within the tumor microenvironment.^24–26^ In the study, to overcome the PD-1/PD-L1 inhibitory pathway, the autologous DC-CIK cells were in vitro directly incubated with a low-dose of pembrolizumab to generate the PD-1 blockade-activated DC-CIK cells (i.e., PD1-T cells). The median dose of pembrolizumab used in the study was 12 mg per infusion, which is much smaller than what was used in clinical practice. The possible excess pembrolizumab in the PD1-T cell product may also affect the host immune cells. However, this systemic effect may be weak, as low dose (0.3–1 mg/kg) of anti-PD-1 antibody has shown little clinical activity.^27,28^ Therefore, we believe the clinical activity of PD1-T cells were primarily due to in vitro pre‐activated DC‐CIK cells, rather than the systemic effects of pembrolizumab on host immune cells. In addition, our previous studies have shown that PD1-T cells were active in several kinds of solid tumor, including CRC.^18,19^ After blocking the PD1/PD-L1 signal pathway with a low dose of pembrolizumab, the cytotoxicity of DC‐CIK cells was enhanced.^18,29^

Previous studies have shown the conceptually mechanisms on the synergistic effects of immune cells combined with chemotherapy. Oxaliplatin used in the study is demonstrated to have immunogenic effects, including induction of immunogenic cancer cell death and enhancement of effector immune response,^30^ which may lead to tumor antigens released from tumor tissues and increase the sensitivity of malignant cells to immune-mediated cytotoxic activity.^31^ On the other hand, similar to the antitumor mechanism exhibited by CIK/DC-CIK cells, PD1-T cells can effectively kill tumor cells through granzyme and perforin-mediated cytotoxic lysis after an MHC-independent tumor recognition,^18,32^ which is different from the anti-tumor mechanism of chemotherapeutic drugs. Meantime, antiangiogenic therapy with bevacizumab could normalize tumor vasculature to let cytotoxic T-cell circulate into tumor cells.^33,34^ These findings indicate that the addition of immune cells to XELOX plus bevacizumab can improve the efficacy of chemotherapy.

The clinical issue underlying the trial holds greater clinical relevance within the subgroup of 173 patients with pMMR CRC. Although PD-1/PD-L1 inhibitors can be beneficial for patients with dMMR mCRC, immune checkpoint blockade (ICB) monotherapy is ineffective in patients with pMMR.^10^ Differing from the ICB therapy that restore the existing immunoreaction by targeting the tumor-induced immune deficiency, adoptive cell immunotherapy directly infiltrated into the pMMR mCRC that is considered a “cold tumor”.^9,12,20^ In the study, patients with pMMR mCRC had significantly survival benefits from the addition of PD1-T cells to chemotherapy. The immunotherapy group exhibited a 38% lower risk of disease progression compared to the control group. Similarly, two recent clinical trials named AtezoTRIBE and MEDITREME also observed the clinical benefit from the addition of ICB therapy to chemotherapy in pMMR/microsatellite stable (MSS) mCRC patients.^35,36^ However, the clinical benefit was somewhat small, with only 1.5 month increase in median PFS in the AtezoTRIBE study, while the magnitude of clinical benefit was difficult to determine due to its single-arm design in the MEDITREME study.^35,36^ Therefore, a phase 3 study was needed to provide additional validation for the therapeutic efficacy of combining ICB therapy with chemotherapy in untreated pMMR/MSS mCRC patients. Nevertheless, the two studies, together with ours, suggest that chemotherapy may promote tumor immunogenicity and enhance the anti-tumoral effect of immunotherapy. Another clinically relevant question is whether there is an association between liver metastases and the effectiveness of ICB therapy in patients with mCRC. Unlike previous studies that reported resistance to ICB therapy among mCRC patients with liver metastases,^20,37–39^ the present study demonstrated that PD1-T cell therapy could provide benefits for patients with liver metastases. This difference may be partly explained by the decreased T-cell infiltration in tumors of patients with liver metastases,^39,40^ while adoptive cell immunotherapy may increase the T-cell infiltration. It would be intriguing to explore the impact of PD1-T cell therapy on pMMR mCRC patients with liver metastases in future clinical study.

This study demonstrates that combining PD1-T cells with XELOX plus bevacizumab has a similar safety profile to previous studies involving CIK cells combined with FOLFOX4 chemotherapy,^17^ as well as CIK cells combined with GP (gemcitabine and cisplatin) chemotherapy in non-small-cell lung cancer.^41^ These findings suggested that the addition of T cell therapy did not further increase the risk of TRAEs compared to the chemotherapy alone. The incidences of TRAEs, grade 3 or above TRAEs, serious adverse events (SAEs), and TRAEs leading to treatment discontinuation were comparable between the two groups. The most frequently occurring grade 3 or above TRAEs in both groups were hematologic toxicity, hand-foot syndrome, diarrhea, and allergy, which is mainly attributed to XELOX plus bevacizumab chemotherapy. There was no significant difference in the incidence of grade 3 or higher hematologic and non-hematologic toxicity between the two groups, and the observed incidence is consistent with previous clinical studies.^42^

Our study has certain limitations. First, at the beginning of this study, the initial treatment choices for patients with dMMR mCRC were chemotherapy plus bevacizumab or cetuximab; thus, the MMR status was not used as one of the inclusion criteria, and about 12% of patients missed the MMR detection. The trial was not powered for analyzing the effect of PD1-T cell therapy on this dMMR population. Second, PD1-T cells were manufactured in a Good Manufacturing Practice-certified facility of each study centers, which may yield potential variability in the PD1-T cell agent, although standard operating procedures under strict quality control and assurance were trained before beginning this trial. Third, the patient randomization allocation procedure was conducted manually, which is more likely to make allocation error than using a central telephone-in or web-based random system. Fourth, the primary endpoint was not centrally reviewed. Finally, potential efficacy-related biomarker tests remain to be analyzed in future studies.

In conclusion, our data indicate that PD1-T cells in combination with XELOX plus bevacizumab is deemed safe and demonstrates significantly improved PFS and OS when compared to XELOX plus bevacizumab alone for mCRC patients. We will continue our efforts to identify predictive markers by analyzing the blood samples collected during this study. PD1-T cells combined with XELOX plus bevacizumab may represent a promising new alternative therapeutic option for patients with mCRC and could be practice changing.

## Materials and methods

### Patients

Patients aged between 18 and 75 years old, with histologically or cytologically confirmed mCRC and at least one measurable disease lesion as per RECIST version 1.1 criteria, progressive disease after surgery or an initial unresectable lesion, and no prior systemic therapy for metastatic disease were deemed eligible. Patients who had undergone previous adjuvant chemotherapy for CRC should have completed treatment at least 6 months before randomization. Other eligibility criteria consisted of an Eastern Cooperative Oncology Group performance status score ranging from 0 to 2, a minimum estimated life expectancy of 3 months, and adequate hepatic, renal, and hematologic functions. Pregnant or nursing women were not considered eligible. Additional exclusion criteria were: prior treatment with monoclonal antibodies targeting PD-1/PD-L1; previous treatment with immune cells; peripheral neuropathy grade ≥ 2 as per the National Cancer Institute Common Terminology Criteria for Adverse Events; previous use of systemic immunosuppressive drugs; other concomitant or previous invasive malignant tumors; active or untreated central nervous system metastasis; other serious life-threatening illness (cardiovascular disease, uncontrolled hypertension, lung disease, or diabetes mellitus); and evidence of bleeding diathesis or significant coagulopathy, ulcer, or serious non-healing wounds.

The study received approval from the institutional ethics committees at each participating site and was conducted in compliance with the Declaration of Helsinki and its subsequent amendments, as well as Good Clinical Practice guidelines. Additionally, all participating patients provided informed consent.

### Trial design and treatment

This phase 3 trial was an open-label, multicenter, randomized, controlled study (ClinicalTrials.gov identifier: NCT03950154) conducted at four medical centers in China (supplementary Table 7). Eligible patients were randomly assigned in a 1:1 ratio to receive autologous PD1-T cell therapy combined with XELOX plus bevacizumab (the immunotherapy group) or XELOX plus bevacizumab alone (the control group) with a block size of four. Randomization was computer generated at the Clinical Trials Center of Sun Yat-sen University Cancer Centre and stratified by treatment center. Sequentially numbered opaque sealed envelopes that contained the details of the random allocations were used to maintain allocation concealment. After eligible patients signed the informed consent, study nurses or clinical research coordinators at each center were responsible for opening the envelopes sequentially and allocated patients to their respective interventions. Both patients and investigators were aware of their assigned treatments and not masked during the study.

Patients assigned to the control group were administered first-line induction therapy with XELOX plus bevacizumab, which included an intravenous infusion of oxaliplatin (130 mg/m^2^) and bevacizumab (7.5 mg/kg) on day 1, along with oral capecitabine (1000 mg/m^2^ twice daily) on days 1–14. In contrast, patients assigned to the immunotherapy group underwent first-line induction therapy using the same XELOX plus bevacizumab regimen as the control group but also received autologous PD1-T cell (about 1 × 10^10^) infusion on day 17. The treatment cycles were administered every 3 weeks for a maximum of six cycles in both groups (supplementary Fig. 2). Then, both groups received maintenance treatment with capecitabine and bevacizumab until disease progression, occurrence of intolerable adverse events, or upon patient requested or the physician decided that therapy should be withdrawn (supplementary Fig. 2). Crossover between treatment groups was not permitted. Dose reduction of chemotherapy was determined by the investigators in accordance with established clinical practice. In order to manage toxic effects, treatment interruption of bevacizumab or PD1-T cells was allowed; however, dose reduction of these agents was not permitted. During the maintenance treatment period, if one of the regimen components was temporarily or permanently suspended due to toxicity, treatment could be continued with the remaining components.

Patients in the immunotherapy group underwent leukapheresis once at least one day before starting treatment to obtain autologous peripheral blood mononuclear cells (PBMCs), which were then cryopreserved in liquid nitrogen. For the six cycles of PD1-T cell infusion, the PD1-T cells were prepared fresh for each cycle from the frozen PBMCs. The PD1-T cells were manufactured according to the methods described in our previous studies.^18,19^ To begin, autologous DC-CIK cells were prepared following our previously established procedures.^43^ In brief, PBMCs were subjected to density gradient centrifugation and were then cultured in X-VIVO 15 medium (Longza) for one hour. Subsequently, the suspended monocytes were obtained to induce CIK cells by adding 1000 µ/ml recombinant human IFN-γ (Clone-gamma, Shanghai Clone Company), along with 100 ng/ml anti-human CD3 monoclonal antibody (R&D Systems), 100 µ/ml IL-1α (Life Technologies) and 1000 U/ml IL-2 (Beijing Sihuan) for the initial 24 h. Adherent monocytes were incubated in X-VIVO 15 serum-free medium supplemented with 30 ng/ml of IL-4 (R&D Systems) and 1000 µ/ml of GM-CSF (Xiamen Amoytop) to induce differentiation into immature DCs, which were subsequently matured using 10 ng/ml of TNF‐α (R&D Systems) on the sixth day. CIK cells were then mixed with the mature DCs at a ratio of 20:1 and cultured in fresh medium containing IL-2 at a concentration of 1000 U/ml for an additional 7 days to induce the formation of DC-CIK cells. Next, on day 14, the autologous DC-CIK cells were assessed for quantity, cell viability, phenotype, and possible presence of bacteria, fungi, and endotoxins. Before cell infusion, the autologous DC-CIK cells were then incubated ex vivo with a humanized IgG4 anti-PD-1 antibody (pembrolizumab, 1 µg/10^6^ cells) for 30–40 min in a thermostat set at 37 °C, referred to as PD1-T cells, and finally transferred to patients.

### Assessments

Tumor assessments were conducted within 28 days of starting study treatment and then repeated every two treatment cycles thereafter. Clinical response was evaluated based on the RECIST criteria, version 1.1, by an independent experienced radiologist at each site, who was unaware of the group assignment. Patients received survival follow-up every 3 months to evaluate clinical outcomes, including subsequent treatment, toxicity, and overall survival until either patient death or the data cutoff date (September 9, 2022), whichever occurred first. Adverse events (AEs), which were defined and graded in accordance with the Common Terminology Criteria for Adverse Events, version 4.03, were assessed from the initiation of the study drug administration until at least 30 days following the last dose of the study drug.

### Endpoints

The primary endpoint of this study was PFS, which was defined as the duration from randomization to the first documented occurrence of disease progression or to death from any cause. The secondary endpoints included OS, objective response rate (ORR), disease control rate (DCR), the duration of response (DOR), and safety. OS referred to the time from the date of randomization until death from any cause. ORR represented the proportion of patients with a confirmed complete (CR) or partial response (PR). DCR indicated the proportion of patients with best response of CR, PR or stable disease (SD). DOR denoted the duration between first documented complete or partial response and subsequent disease progression or death.

### Statistical analyses

The calculation of the sample size was predicated on the primary endpoint of PFS. Previous studies suggested that the control group would have a median PFS of 10.0 months.^4–7^ The expected median PFS for the immunotherapy group is 16.5 months, with a hazard ratio (HR) of 0.606. The required sample size to detect a 40% risk reduction with 80% power using a log-rank test at a two-sided significance level of 0.05 was 179 patients, assuming enrollment over a period of two years and follow-up for another one year. Considering a dropout rate of 10%, at least 99 patients per group and a total study population of 198 patients were deemed necessary as calculated by PASS version 15 (NCSS, LLC).

Efficacy analyses were conducted in the intention-to-treat population, while safety assessment focused on the as-treated population who received at least one dose of study treatment. Patients were censored at their last imaging assessment for PFS and at their last known alive status for OS. In cases where a postbaseline tumor evaluation was not performed, the best overall response of the patient was considered non-evaluable. The Kaplan-Meier method was used to estimate survival curves. Between-group differences in PFS and OS were evaluated as primary analysis using stratified log-rank test (stratified by treatment center). Hazard ratio (HR) for PFS and OS, along with 2-sided 95% confidence interval (CI) were calculated with a stratified Cox proportional-hazards model. The assumption of proportional hazards was evaluated by both graphical and analytic methods. To investigate the effect of treatment discontinuation, sensitivity analyses were performed for PFS. Additionally, prespecified subgroup analysis was performed using univariate Cox proportional hazards regression models to assess the heterogeneity between the immunotherapy and the control subgroups. A Cox model was used to calculate a test for interaction (*p* interaction), which included the treatment group, subgroup variable, and their interaction term. Objective response and disease control were reported with 95% CIs, which was calculated using the Clopper-Pearson method, and the comparisons between the groups were conducted using the Pearson χ2 test. Chi-square test and Fisher exact test were employed to compare binary variable. Statistical analyses in this study were conducted utilizing SPSS (version 22) and GraphPad Prism (version 9.5.1).

### Supplementary information

Supplementary Materials

## Data Availability

The data that support the findings of this study are available from the corresponding author upon reasonable request.
