# Supplementary material for: XELOX (capecitabine plus oxaliplatin) plus bevacizumab (anti-VEGF-A antibody) with or without adoptive cell immunotherapy in the treatment of patients with previously untreated metastatic colorectal cancer: a multicenter, open-label, randomized, controlled, phase 3 trial
Source: Signal Transduct Target Ther. 2024 Apr 3;9:79. doi: 10.1038/s41392-024-01788-2 (PMC10987514; doi:10.1038/s41392-024-01788-2)
Supplement: Supplementary file 1 — Supplementary Materials [file 41392_2024_1788_MOESM1_ESM.docx]

Supplementary Materials for

XELOX (capecitabine plus oxaliplatin) plus bevacizumab (anti-VEGF-A antibody) with or without adoptive cell immunotherapy in the treatment of patients with previously untreated metastatic colorectal cancer: a multicenter, open-label, randomized, controlled, phase 3 trial

Qiu-Zhong Pan, Jing-Jing Zhao, Liang Liu, Dong-Sheng Zhang, Li-Ping Wang, Wen-Wei Hu, De-Sheng Weng, Xiang Xu, Yi-Zhuo Li, Yan Tang, Wei-Hong Zhang, Jie-Yao Li, Xiao Zheng, Qi-Jing Wang, Yong-Qiang Li, Tong Xiang, Li Zhou, Shuang-Ning Yang, Chen Wu, Rong-Xing Huang, Jia He, Wei-Jiao Du, Lu-Jun Chen, Yue-Na Wu, Bin Xu, Qiong Shen, Yi Zhang, Jing-Ting Jiang, Xiu-Bao Ren, and Jian-Chuan Xia

Correspondence to: [xiajch@mail.sysu.edu.cn](mailto:xiajch@mail.sysu.edu.cn); [renxiubao@tjmuch.com](mailto:renxiubao@tjmuch.com); jiangjingting@suda.edu.cn; [yizhang@zzu.edu.cn](mailto:yizhang@zzu.edu.cn).

**This PDF file includes:**

Table S1 to S7

Figure S1 to S2

**Table S1. Treatment exposure by treatment subgroup over the induction period**

| **Treatment measure** | **Immunotherapy group**  **(n = 100)** | **Control group**  **(n = 102)** |
| --- | --- | --- |
| Median (range) No. of cycles received | 6 (1 – 6) | 6 (1 – 6) |
| Median cumulative dose received |  |  |
| Oxaliplatin, mg/m^2^ | 769.2 | 765.1 |
| Capecitabine, mg/m^2^ | 11346.8 | 11107.9 |
| Bevacizumab, mg/kg | 44.3 | 44.1 |

**Table S2. Subsequent anti-cancer therapy in intent-to-treat population after the study treatment**

| **Treatment** | **Immunotherapy group**  **(n = 100)**  **N (%)** | **Control group**  **(n = 102)**  **N (%)** | **Total**  **(n = 202)**  **N (%)** |
| --- | --- | --- | --- |
| Patients with at least one subsequent anti-cancer therapy | 56 (56.0%) | 66 (64.7%) | 122 (60.4%) |
| FOLFIRI+Bevacizumab | 21 (21.0%) | 29 (28.4%) | 50 (24.8%) |
| FOLFIRI+Cetuximab | 3 (3.0%) | 2 (2.0%) | 5 (2.5%) |
| FOLFIRI | 1 (1.0%) | 3 (2.9%) | 4 (2.0%) |
| Irinotecan+Capecitabine+Bevacizumab | 1 (1.0%) | 3(2.9%) | 4 (2.0%) |
| Irinotecan+Bevacizumab | 2 (2.0%) | 2 (2.0%) | 4 (2.0%) |
| Irinotecan+Raltitrexed+Bevacizumab | 1 (1.0%) | 1 (1.0%) | 2 (1.0%) |
| Irinotecan+Tegafur+Bevacizumab | 2 (2.0%) | 1 (1.0%) | 3 (1.5%) |
| Irinotecan+Tegafur+Sindillimab | 1 (1.0%) | 0 | 1 (0.5%) |
| Irinotecan+Capecitabine | 0 | 1 (1.0%) | 1 (0.5%) |
| XELOX+Bevacizumab | 1 (1.0%) | 2 (2.0%) | 3 (1.5%) |
| FOLFOX+Cetuximab | 1 (1.0%) | 0 | 1 (0.5%) |
| Capecitabine+Bevacizumab | 3 (3.0%) | 2 (2.0%) | 5 (2.5%) |
| Capecitabine | 1 (1.0%) | 0 | 1 (0.5%) |
| Oxaliplatin+Raltitrexed+Bevacizumab | 0 | 2 (2.0%) | 2 (1.0%) |
| Encorafenib+Cetuximab | 0 | 1 (1.0%) | 1 (0.5%) |
| 5-FU+Cetuximab | 0 | 1 (1.0%) | 1 (0.5%) |
| 5-FU+Bevacizumab | 1 (1.0%) | 0 | 1 (0.5%) |
| Tegafur+Docetaxel | 2 (2.0%) | 0 | 2 (1.0%) |
| Fruquintinib | 2 (2.0%) | 2 (2.0%) | 4 (2.0%) |
| Albumin-bound paclitaxel+Lobaplatin | 1 (56.0%) | 0 | 1 (0.5%) |
| Nivolumab | 1 (56.0%) | 0 | 1 (0.5%) |
| Ablation therapy | 0 | 2 (2.0%) | 2 (1.0%) |
| Surgery | 6 (6.0%) | 10 (9.8%) | 16 (7.9%) |
| Radiotherapy | 2 (2.0%) | 0 | 2 (1.0%) |
| Traditional herbal therapy | 3 (3.0%) | 2 (2.0%) | 5 (2.5%) |

FOLFIRI, 5-fluorouracil/leucovorin + irinotecan; FOLFOX, 5-fluorouracil/leucovorin + oxaliplatin; XELOX, capecitabine + oxaliplatin

**Table S3. Summary of injected PD1-T cell agent**

|  | **Immunotherapy group**  **(n = 100)** |  |
| --- | --- | --- |
| **Cumulative total cell count received, × 10^10^** |  |  |
| Mean ± SD | 6.1 ± 2.2 |  |
| Median (range) | 7.0 (0-9.9) |  |
| **CD3^+^ cell, %** | |  |
| Mean ± SD | | 95.6 ± 3.6 |
| Median (range) | | 96.9 (80.2-99.6) |
| **CD3^+^CD8^+^cell, %** | |  |
| Mean ± SD | | 66.8 ± 16.3 |
| Median (range) | | 69.2 (6.1-95.9) |
| **CD3^+^CD4^+^cell, %** | |  |
| Mean ± SD | | 28.1 ± 15.6 |
| Median (range) | | 25.7 (1.5-83.8) |
| **CD3^+^CD56^+^cell, %** | |  |
| Mean ± SD | | 17.7 ± 9.1 |
| Median (range) | | 16.4 (1.1-64.3) |
| **CD3^-^CD56^+^cell, %** | |  |
| Mean ± SD | | 3.6 ± 3.6 |
| Median (range) | | 2.3 (0.1-16.1) |
| **Injection times** |  |  |
| 0 | 1 (1.0%) ^a^ |  |
| 1 | 3 (3.0%) |  |
| 2 | 9 (9.0%) |  |
| 3 | 4 (4.0%) |  |
| 4 | 5 (5.0%) |  |
| 5 | 8 (8.0%) |  |
| 6 | 70 (70.0%) |  |
| Total | 513 |  |

^a^ One patient died of obstructive pneumonia after the first cycle of chemotherapy and did not receive any PD1-T cell infusion.

**Table S4. Sensitivity analysis for progression-free survival (PFS) according to the censoring strategy of treatment discontinuation between the two groups.**

| **Type of Analysis** | **Median PFS (months)** | | **HR** | **95% CI** | ***p*** |
| --- | --- | --- | --- | --- | --- |
|  | **The immunotherapy group** | **The control group** |  |  |  |
| Primary analysis of PFS | 14.8 | 9.9 | 0.60 | 0.40-0.88 | 0.009 |
| Patients who discontinued treatment and started new anticancer drug therapy without documented progression were considered PD events in the two groups | 12.7 | 9.2 | 0.62 | 0.44-0.90 | 0.010 |
| Patients who discontinued treatment and started new anticancer drug therapy without documented progression were considered PD events in the immunotherapy group and censored in the control group | 12.7 | 9.9 | 0.69 | 0.47-1.00 | 0.047 |

PFS, progression-free survival; HR, hazard ratio; CI, confidence interval; PD, progressive disease.

**Table S5. Intent-to-diagnose sensitivity analysis for PFS with the mutation status (RAS, BRAF) and MMR status imputed for unavailable patients based on observed prevalence**

|  | **HR (95% CI) ^a^** | ***p* for interaction** |
| --- | --- | --- |
| RAS available ^b^ | 1.51 (0.65-3.50) | 0.339 |
| RAS available + imputed | 1.45 (0.64-3.30) | 0.376 |
| RAF available | 1.15 (0.27-4.95) | 0.849 |
| RAF available + imputed | 1.04 (0.24-4.49) | 0.963 |
| MMR available | 0.18 (0.01-3.13) | 0.237 |
| MMR available + imputed | 0.27 (0.02-4.43) | 0.355 |

PFS, progression-free survival; HR, hazard ratio; CI, confidence interval.

^a^ HR is from Cox proportional hazards model; *p* value is for the same HR, and was computed using the Cox proportional hazards regression model with the treatment group, the subgroup variable and their interaction as covariates; columns for available + imputed display the mean and 95% CI of parameter estimates from 5,000 simulated trials.

^b^ Primary analysis limited to patients available for the mutation status (RAS, BRAF) and MMR status.

**Table S6. All-cause serious adverse events in the safety population, according to treatment group.**

|  | **Immunotherapy group**  **(n = 100)** | **Control group**  **(n = 102)** |
| --- | --- | --- |
| Any event | 13 (13.0%) | 15 (14.7%) |
| Gastrointestinal obstruction | 3 (3.0%) | 3 (3.0%) |
| Pneumonia | 2 (2.0%) | 1(1.0%) |
| Pyrexia | 1 (1.0%) | 2 (2.0%) |
| Diarrhea | 1 (1.0%) | 3 (3.0%) |
| Intestinal perforation | 1 (1.0%) | 1 (1.0%) |
| Gallstone | 1 (1.0%) | 0 |
| Hyperbilirubinemia | 1 (1.0%) | 0 |
| Urinary tract infection | 1 (1.0%) | 0 |
| Hypocortisolism | 1 (1.0%) | 0 |
| Anastomotic fistula | 1 (1.0%) | 0 |
| Thrombocytopenia | 0 | 1 (1.0%) |
| Multiple organ failure | 0 | 1 (1.0%) |
| Liver dysfunction | 0 | 1 (1.0%) |
| Hypertension | 0 | 1 (1.0%) |
| Arhythmia | 0 | 1 (1.0%) |

**Table S7. Study centers and the number of patients included into the study.**

| **Name of site/city** | **Principal Investigator** | **Number of patients** **recruited** |
| --- | --- | --- |
| Sun Yat-Sen University Cancer Center, Guangzhou | Jian-Chuan Xia | 92 |
| Tianjin Medical University Cancer Institute and Hospital, Tianjin | Xiu-Bao Ren | 36 |
| the First Affiliated Hospital of Zhengzhou University, Zhengzhou | Yi Zhang | 37 |
| the Third Affiliated Hospital of Soochow University, Changzhou | Jing-Ting Jiang | 37 |


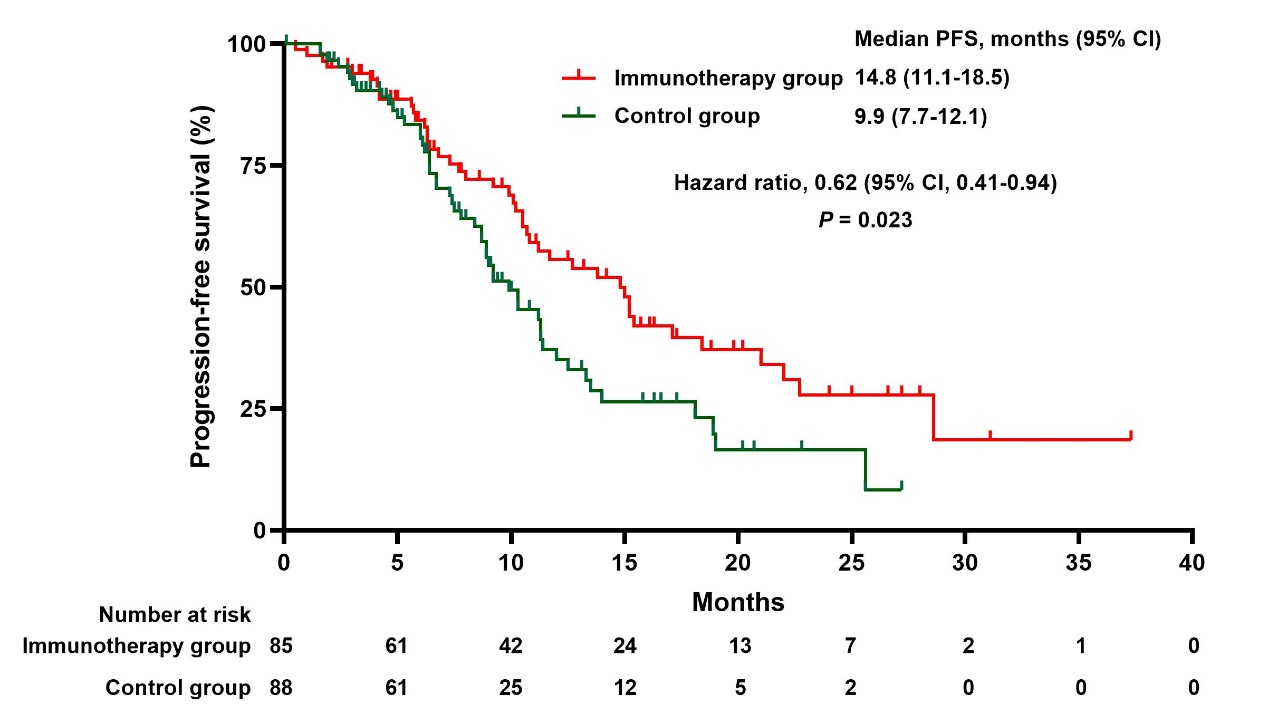


**Figure S1. Progression-free survival (PFS) of patients with the DNA mismatch repair–proficient (pMMR) metastatic colorectal cancer.**


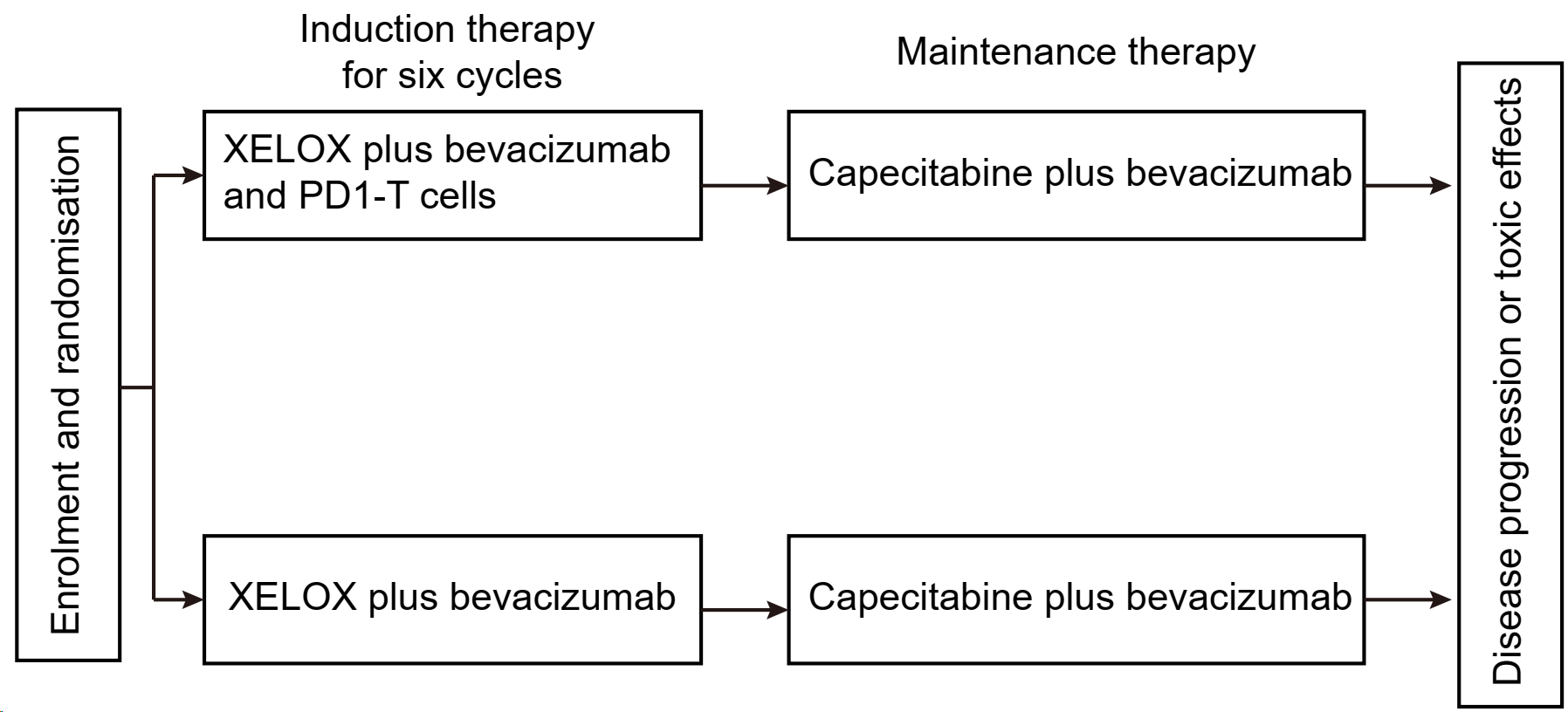


**Figure S2. Treatment schedules.**

XELOX = oxaliplatin (130 mg/m^2^, d1) plus capecitabine (1000 mg/m^2^ twice daily, day 1 to day 14), every 3 weeks; PD1-T cells = PD-1 blocked-activated DC-CIK cells.
